# Preparation of Ag NPs and Its Multifunctional Finishing for Cotton Fabric

**DOI:** 10.3390/polym13081338

**Published:** 2021-04-19

**Authors:** Jionglin Zhu, Hong Li, Yu Wang, Yusu Wang, Jun Yan

**Affiliations:** Department of Textile and Material Engineering, Dalian Polytechnic University, Dalian 116034, China; zhujionglin90@163.com (J.Z.); dpuwangyu@163.com (Y.W.); fz172wys@163.com (Y.W.); yanjun@dlpu.edu.cn (J.Y.)

**Keywords:** honeysuckle extracts, Ag NPs, polycarboxylic acid, multifunctional properties, washability, cotton fabrics

## Abstract

To explore the combination of silver nanoparticles (Ag NPs) prepared in a green manner with cotton fabrics and the washing durability of the fabric after the combination. In this paper, the natural material, honeysuckle extract, was used as a reducing agent to prepare the Ag NPs’ solution. The structure and size of Ag NPs were analyzed using ultraviolet–visible spectrophotometry (UV–vis), transmission electron microscopy (TEM), dynamic light scattering (DLS), X-ray powder diffraction (XRD), and Fourier transform infrared (FT-IR) spectroscopy characterization. The results showed that Ag^+^ was successfully reduced to Ag^0^ by the honeysuckle extract, the particle size was about 10.59 nm, and the potential was −42.9 mV, so it had strong electrostatic repulsion and good stability. Meanwhile, it was found that the synthesized Ag NPs were well coated by the honeysuckle extract, so they would not aggregate. Then, the cotton fabric was finished with Ag NPs’ solution by the dipping method using a complex of polymaleic acid (PMA) and citric acid (CA) as a cross-linking agent to fix Ag NPs on the cotton fabric. The structures of cotton fabrics before and after finishing were characterized using FT-IR, scanning electron microscopy (SEM), XRD, X-ray photoelectron spectroscopy (XPS), and thermogravimetric (TG) analysis, and the multifunctional properties of the finished cotton fabrics were explored by measuring the antibacterial rate, the wrinkle recovery angle (WRA), and the UV protection factor (UPF) value. The results show that Ag NPs were successfully loaded onto cotton fabric, and the PMA + CA compound was successfully cross-linked to the fabric. The cross-linked Ag NPs’ cotton fiber was rougher than that before cross-linking, and its TG stability improved. The PMA + CA compound fixed Ag NPs on the cotton fabric through chemical bonds, so it still had a 99% antibacterial effect against Escherichia coli (*E. coli*) and Staphylococcus aureus (*S. aureus*) after 50 washings. Compared with unfinished cotton fabric, the UPF value and WRA of the cross-linked Ag NPs cotton increased by 34.09 and 98°, respectively, and its color did not change much.

## 1. Introduction

Cotton fabric is a type of natural porous fabric with good wearing properties, such as moisture absorption, breathability, and skin-friendliness [1,2]. However, cotton fabrics have problems such as being easy to wet, easy to stain with oil, and easy to wrinkle, and with poor UV resistance performance. Bacteria are prone to breed on the surface of wet and oily cotton fabrics, leading to an increased risk of illness for users. Wrinkling is one of the most important shortcomings of cotton fabrics. Cotton fabrics can promote the internal movement of polymer chains under moist conditions, thereby removing the hydrogen bonds existing in the cellulose structure. Later, new hydrogen bonds are reorganized in new positions, and obvious folds appear on the surface of the fabric [3,4]. At the same time, cotton fabrics have poor UV blocking performance, which accelerates the skin aging of users and even induces skin cancer, which will also bring hidden dangers to the health of users. Therefore, the research and development of cotton-based textiles with long-term antibacterial properties and other functions is particularly important.

Ag NPs are a type of nanoscale elemental silver, which is an efficient and broad-spectrum inorganic antibacterial finishing agent [5]. In recent years, with their unique antibacterial properties, Ag NPs have had important research and application values in the fields of medicine and health, detergents, clothing, etc., and have been widely studied by scholars at home and abroad [6,7,8]. The synthesis technology of Ag NPs is becoming more and more mature, however, the reducing agents used in it, such as sodium borohydride, increase environmental or biological hazards [9,10]. Therefore, from the perspective of environmental protection and large-scale production, it is necessary to explore an efficient, simple, green, and nontoxic method for preparing Ag NPs. At present, the biosynthesis method using plant extracts or microorganisms has become a simple and feasible method to replace physical and chemical synthesis methods [11]. The basic raw materials used in this method are natural substances, and their functional biomolecules can effectively reduce metal ions [12]. It is not only low-cost, nonpolluting, environmentally friendly, and suitable for large-scale preparation, but also the reducing agent and capping agent used in the synthesis process are also environmentally friendly [13].

Many medicinal plants contain large amounts of antioxidants, such as polyphenols, flavonoids, and amide compounds [14,15]. These compounds contain reducing hydroxyl groups, which can reduce silver ions to elemental silver. Because of the high electronegativity of the oxygen in the hydroxyl group, they can prevent the agglomeration of Ag NPs and control the size of Ag NPs in the nanometer range, acting as a protective agent [16]. Honeysuckle is a Chinese herbal medicine plant with abundant availability and wide distribution. It contains many different polyphenols, such as chlorogenic acid, flavonoids, and glycosides. The molecular structure of these substances contains a large number of hydroxyl groups, making them highly reducing. It can also protect Ag NPs from agglomeration. Therefore, honeysuckle extract is a natural reducing agent and protective agent with a strong reducing ability.

An important property of functional cotton fabrics is washing resistance. Cotton fabric finished with nanoparticles has good functionality, but after several washings, the functionality of the cotton fabric decreases, mainly due to the poor absorption performance of the cotton fabric to the nanoparticles and easy washing off. To enhance the absorption performance of the cotton fabric to nanoparticles, a cross-linking agent can be added to the cotton fabric. The principle is to introduce reactive groups such as –SH, –NH2, or –COOH into the cotton fabric through graft modification, and establish chemical bonds or other forms of firm bonding between these groups and nanoparticles to introduce nanoparticles into cotton, thereby giving cotton fabrics long-term functionality [17,18,19]. Among the cross-linking agents of polycarboxylic acids, 1,2,3,4-butanetetracarboxylic acid (BTCA) is more effective than cheap CA and PMA as a crosslinker for cotton [20]. Research indicates that the reaction between CA and the anhydride intermediates of PMA transforms CA from a trifunctional crosslink to a tetrafunctional acid with much higher effectiveness in the curing process and creates a synergic effect [21].

Presently, some progress has been made in the preparation of Ag NPs from plant extracts, loading Ag NPs into fabrics, and improving the washable functionality of fabrics, but there are few reports on combining the three. Therefore, based on the research and analysis of relevant literature, the research idea of this paper was to prepare Ag NPs with honeysuckle extract, load Ag NPs on cotton fabric by a dipping method, and use the PMA + CA compound as a cross-linking agent, sodium hypophosphite (SHP) as a catalyst for modification, to make the fabric multifunctional with durable properties. The morphology and structure of the finished cotton fabrics were characterized, the antibacterial, anti-ultraviolet, anti-wrinkle, and other wearing properties of the finished cotton fabrics were studied, and the relationship between the color change of the finished fabric, the silver content, and the number of washings are discussed, to obtain green, efficient, environmentally friendly, long-term antibacterial and anti-ultraviolet multifunctional textiles.

## 2. Materials and Methods

### 2.1. Materials

The scoured and bleached cotton woven fabrics (14.0 tex × 13.2 tex, warp count: 270/10 cm; weft count: 220/10 cm; Ends/tex: 14.0; Picks/tex: 13.2, Foshan Shihong Textile Co., Ltd., Shandong, China). Silver nitrate (AgNO_3_, 99.8%, analytical grade, Shengben Technology Co., Ltd., Zhejiang, China). Sodium chloride (NaCl, analytical grade, Hubei Guangao Biotechnology Co., Ltd., Hubei, China). Honeysuckle extract (80 mesh, chlorogenic acid: 50%, Shenzhen Simeiquan Biotechnology Co., Ltd., Shenzhen, China). Beef extract, peptone, nutrient agar (biological agents, Shanghai Guduo Biological Technology Co., Ltd., Shanghai, China). CA, PMA (Mw = 400–800), and SHP were of analytical grade and purchased from Tianjin Komil Chemical Reagent Co., Ltd., Tianjin, China.

### 2.2. Synthesis of Ag NPs

For all experiments, the source of silver was silver nitrate. Honeysuckle extract (1 g) was dissolved in 50 mL deionized water, centrifuged at 6000 r/min for 10 min, and the supernatant was collected for use as honeysuckle extract solution. Ag NPs were prepared using 3 mL honeysuckle extract solution and 0.5 mL of an aqueous solution of 0.1 mol/L silver nitrate solution in a 250 mL Erlenmeyer flask and heated in an oil bath at 130 °C for 15 min. The color of the solution changed rapidly from yellow to brown, which visually illustrates the generation of Ag NPs.

### 2.3. Preparation of Ag NPs Finished Cotton

First, the cotton was pretreated (a piece of cotton was washed three times with ethanol and deionized water) to remove impurities and organic matter from the surface, and dried for later use. The pretreated cotton fabric was put into the Ag NPs’ solution (bath ratio 1:30), and the Ag NPs cotton fabric was impregnated with stock for 60 min at a temperature of 50 °C. Finally, the treated cotton was dried at 50 °C to constant weight to obtain Ag/Cotton.

Second, the pretreated cotton fabric was fully immersed in a prepared solution of Ag NPs, CA (3%), PMA (3%), and SHP (3%) at a bath ratio of 1:30 using an “impregnation” method, and stirred at a temperature of 50 °C for 60 min. Then, it was dried at 70 °C for 30 min and cured at 160 °C for 2 min to obtain the PMA–CA–Ag/Cotton fabric. After the cotton fabric was removed, it was rinsed with deionized water several times. Then, the cotton fabric was treated with 0.1 mol/L NaOH solution for 3 min at room temperature to convert the unreacted carboxylic acid into carboxylate anion. Finally, the PMA–CA–Ag/Cotton was washed with deionized water and dried at low temperature to constant weight.

### 2.4. Characterization of Ag NPs and Finished Fabrics

#### 2.4.1. UV–vis Spectroscopy Analysis

The Ag NPs were measured with a UV spectrophotometer (UV-6000PC, Shanghai, China) and poured into a cuvette, deionized water was used as a reference sample, the measurement range was 300–700 nm, the measurement interval was 0.5 nm, and the UV–vis spectra were obtained.

#### 2.4.2. Transmission Electron Microscope Analysis

Transmission electron microscope (TEM) images of the Ag NPs were obtained using a TEM (LEAP 4000X HR, Shenzhen, China). The Ag NPs’ solution was dropped on the copper grid, and after it was fully dried, it was placed in a TEM instrument to observe its particle size and morphology.

#### 2.4.3. DLS Analysis

The particle size and potential of silver nanoparticles were measured using dynamic light scattering (DLS). About 1 mL of Ag NPs’ solution was put into a standard cuvette of the laser particle size analyzer (Nano-ZS, Wuhan, China) to determine its particle size and distribution.

#### 2.4.4. X-ray Powder Diffraction (XRD) Analysis

The Ag NPs, Ag/Cotton, and PMA–CA–Ag/Cotton were tested using an X-ray diffractometer (XRD-6100, Xianggang, China). Test conditions: tube voltage 40 kV, tube current 30 mA, scanning speed: 5°/min, 2θ: 10–70°.

#### 2.4.5. Fourier Transform Infrared (FT-IR) Analysis

The Ag NPs, Ag/cotton, and PMA–CA–Ag/cotton were measured using an infrared spectrometer (FT-IR-650, Tianjin, China) under the conditions of temperature 20 °C and humidity 65% after pressing in a KBr pellet. The wave number range: 4000–400 cm^−1^.

#### 2.4.6. Scanning Electron Microscopy (SEM) Analysis

The surface morphologies of the Ag/Cotton and PMA–CA–Ag/Cotton were observed using a scanning electron microscope (Alpha300S, Xianggang, China). The fabrics were sprayed with gold under the conditions of a temperature of 20 °C and relative humidity of 65%.

#### 2.4.7. X-ray Photoelectron Spectroscopy (XPS) and Thermogravimetric (TG) Analysis

The cotton fabrics were analyzed using an X-ray photoelectron spectrometer (Thermo Scientific, Henan, China) to determine the element valence state. The test vacuum was 2 × 107 Pa, and the step length was 0.1 eV. TG analyses of the Ag/Cotton and PMA–CA–Ag/Cotton were performed using a thermal gravimetric analyzer (TGA-101, Shanghai, China).

#### 2.4.8. Silver Content in Fabric Analysis

The Ag/Cotton and PMA–CA–Ag/Cotton were weighed (50 mg), dissolved in 10 mL of concentrated nitric acid (65% by mass), and the volume was diluted to 100 mL with deionized water, and then the values were measured with an inductively coupled plasma spectrometer (Vista-MPX V, Palo Alto, CA, USA). The Ag band (328 nm) was used to calculate the silver content on cotton fabrics.

#### 2.4.9. The Fabric ∆E Analysis

The Ag/Cotton and PMA–CA–Ag/Cotton were folded into four layers and tested for various color indexes (L*, a*, and b*) using a color measuring and matching instrument (Color-Eye7000A, Shanghai, China), and then the ∆E of each sample was calculated according to formula (1). Test light source: D65 light source, observation angle: 10°.

(1) $$\Delta E=\sqrt{{\left(\Delta L^{*}\right)}^{2}+{\left(\Delta a^{*}\right)}^{2}+{\left(\Delta b^{*}\right)}^{2}},$$

where ∆E is the color difference value, ∆L* is the color difference between the finished sample and the original sample, ∆a* is the red–green difference between the finished sample and the original sample, and ∆b* is the yellow–blue difference between the finished sample and the original sample.

#### 2.4.10. Antibacterial Rate Analysis

*E. coli* (ATCC25922) and *S. aureus* (ATCC6538), the two most representative strains of Gram-negative and Gram-positive bacteria, were selected as test strains, and the antibacterial properties and antibacterial washing resistance of the fabric were tested by referring to the third part of the oscillation method of GB/T 20944.2-2008 standards. The sterilized unfinished cotton, the Ag/cotton and PMA–CA–Ag/cotton were placed in conical flasks, then 50 mL of bacterial solution with a concentration range of 3 × 10^5^~4 × 10^5^ CFU/mL and 0.85% normal saline were added, and then placed in a constant-temperature oscillating incubator at 37 °C for 24 h, the oscillation frequency was 145 r/min. After the oscillation culture was completed, the solution in the conical flask was diluted by a 10-fold gradient dilution method, and then added dropwise into an AGAR culture dish and incubated in a constant-temperature incubator at 37 °C for 24 h. The growth of bacteria was observed in the petri dishes. The final experimental data are the arithmetic averages of three parallel experiments for each group. The bacteriostatic rate was calculated according to Formula (2):

(2) $$Y=\frac{A-B}{A}\times 100\%,$$

where Y is the antibacterial rate, A is the number of colonies of unfinished cotton (CFU/mL), B is the number of colonies of finishing cotton (CFU/mL).

#### 2.4.11. Wash Fastness

Wash fastness tests of finished fabrics were conducted according to the standard titled “Textiles Tests for color fastness-Part C10: Colorfastness to washing with soap or soap and soda”. In this way, the finished fabrics were first immersed in a washing solution containing commercial detergent (5.0 g/L), and the material-to-liquid ratio of the washing solution was 1:50. Next, the fabrics were stirred and allowed to stand at 40 ± 5 °C for 15 min. Finally, the fabrics were rinsed with tap water. This process was repeated 10, 20, 30, 40, and 50 times to obtain fabrics with 10, 20, 30, 40, and 50 washing cycles.

#### 2.4.12. UV and Crease Resistance of Fabrics

The UV resistance of the Ag/Cotton and PMA–CA–Ag/Cotton were measured using a Textile UV Performance Tester (YG(B)912E, Wenzhou, China). The wrinkle recovery angle (WRA) of fabrics was tested using an automatic digital fabric crease elastometer (YG(B)541D-II, Wenzhou, China).

## 3. Results and Discussion

### 3.1. UV–Vis Characterization of Ag NPs

**Figure 1:**
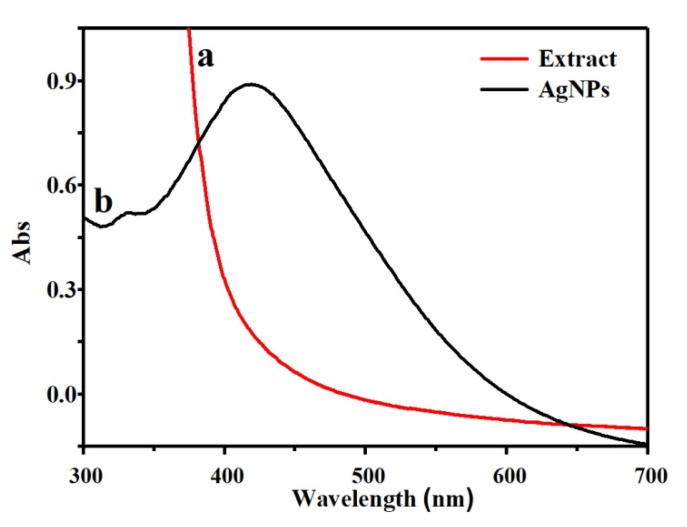
UV-Vis of honeysuckle extract (**a**) and Ag NPs (**b**).

Because of the surface plasmon resonance effect (SPR), Ag NPs usually strongly and obviously absorb light of a specific wavelength, and then show a particular color. The absorption peak at 400–450 nm is the basis of the wavelength of Ag NPs [22], it also forms the SPR absorption phenomenon of spherical or nearly spherical Ag NPs [23,24], so the test first adopted the method of observing the color change of the solution and the UV–vis spectrum of the test solution to determine preliminarily the generation of Ag NPs. Figure 1 shows the color of the solution before and after the reaction and the changes in the UV–vis spectrum. It can be seen that the color of the solution has changed from light yellow before the reaction (the color of honeysuckle extract) to reddish-brown after the reaction (the color of the Ag NPs’ solution), and the UV–vis spectrum shows an obvious symmetrical absorption peak at about 418 nm, according to the SPR effect, it is proved that Ag NPs can be obtained by reducing silver nitrate with honeysuckle extract under appropriate conditions.

### 3.2. TEM Characterization of Ag NPs

The size, shape, and morphology of the synthesized Ag NPs were characterized using TEM.

**Figure 2:**
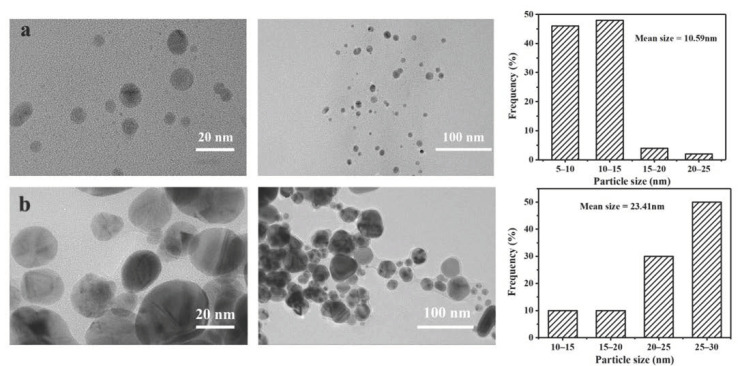
TEM images and particle size distribution of Ag NPs (**a**) and Ag NPs after one month placement (**b**).

It can be seen from Figure 2a that most of the Ag NPs prepared using the honeysuckle extract were approximately spherical and uniformly distributed between 5 and 25 nm, and that the average particle size was about 10.59 nm. There were a large number of Ag NPs and a good dispersion state at the same time. Figure 2b is the surface morphology of Ag NPs after being stored for one month. It can be seen that the particle sizes of Ag NPs were slightly larger, the particle size distribution was between 10 and 30 nm, and the average particle size was about 23.41 nm. This may be because, during this period, a small number of Ag NPs nuclei were still generated, and the honeysuckle extract cannot be coated on the surface of each Ag NP in time, so that the Ag NPs nuclei were agglomerated, the particle size became larger, and the distribution range of the diameter of the particles became larger. It can also be seen that the Ag NPs after placement still had a good dispersion state because most of the Ag NPs were relatively stable and would not agglomerate, so the Ag NPs prepared using honeysuckle extract have good stability [25].

### 3.3. Laser Particle Size Measurement and Potential Analysis of Ag NPs

**Figure 3:**
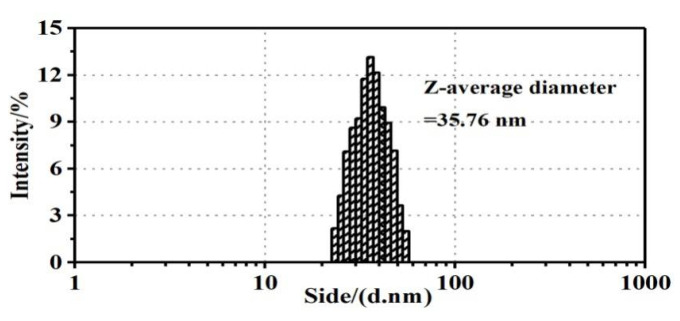
Particle size distribution of Ag NPs.

Figure 3 shows the particle size distribution of Ag NPs in solution. It can be seen that the particle size distribution of Ag NPs basically shows a normal distribution trend, indicating that the particle size distribution was relatively uniform and concentrated. It is also worth noting that the average particle size of Ag NPs measured using a laser particle sizer was much larger than that measured using TEM, which may be caused by the following factors: first, the particle size measured using the DLS method was not the true size of the nanoparticle, but was the hydraulic diameter of the nanoparticle in the solution; second, the plant organic components adsorbed on the surface of the Ag NPs caused the high test results; third, due to the extremely high surface energy of Ag NPs, they could adsorb water molecules to form a film of water molecules, which affected the particle size test results [26,27].

**Figure 4:**
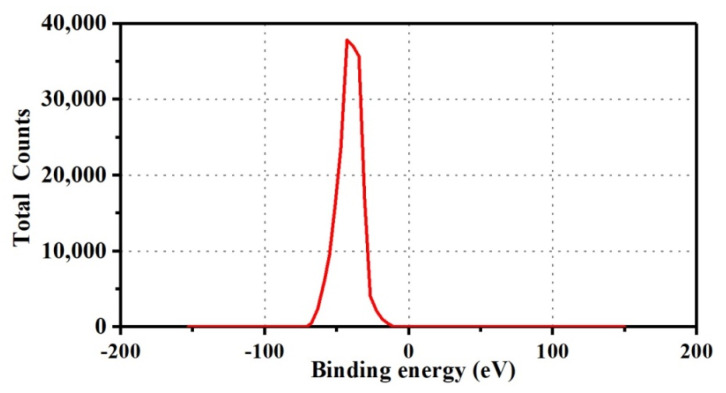
Potential diagram of Ag NPs.

Zeta potentials of Ag NPs were measured, and the results are shown in Figure 4. The zeta potential of Ag NPs was −42.9 mV, indicating that there was a strong electrostatic repulsion between the Ag NPs, which confirmed the high stability of the Ag NPs. The negative potential value might be due to the presence of biological organic components in the extract as a capping agent, which played a crucial role in the stability of Ag NPs.

### 3.4. XRD Characterization of Ag NPs

**Figure 5:**
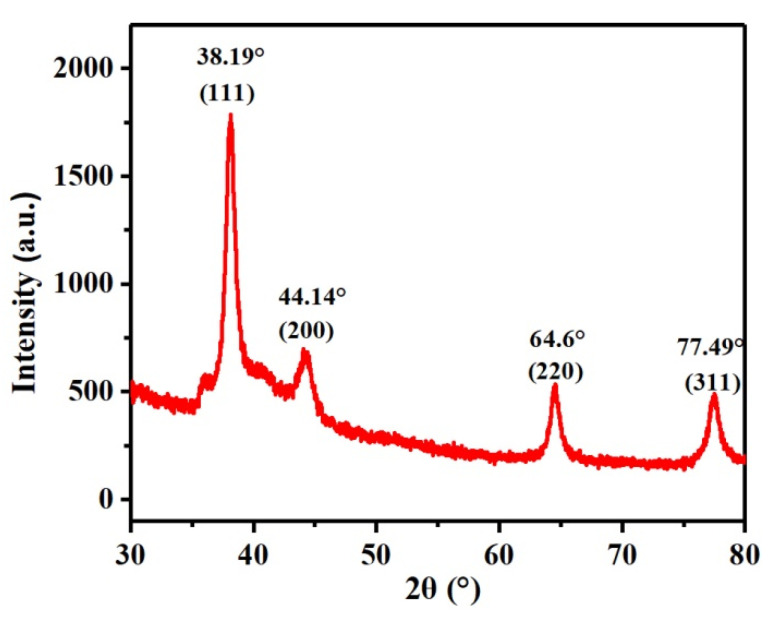
XRD pattern of Ag NPs.

Figure 5 shows the XRD pattern of Ag NPs prepared using honeysuckle extract. It can be seen that the positions of the diffraction peaks of 38.12, 44.31, 64.45, and 77.41° correspond to the crystal surface of silver (111), (200), (220), and (311), respectively, indicating that the Ag NPs were successfully prepared using honeysuckle extract as the reducing agent [28,29]. The XRD pattern shows that the product is pure Ag NPs with high crystallinity.

### 3.5. FT-IR Characterization of Ag NPs

**Figure 6:**
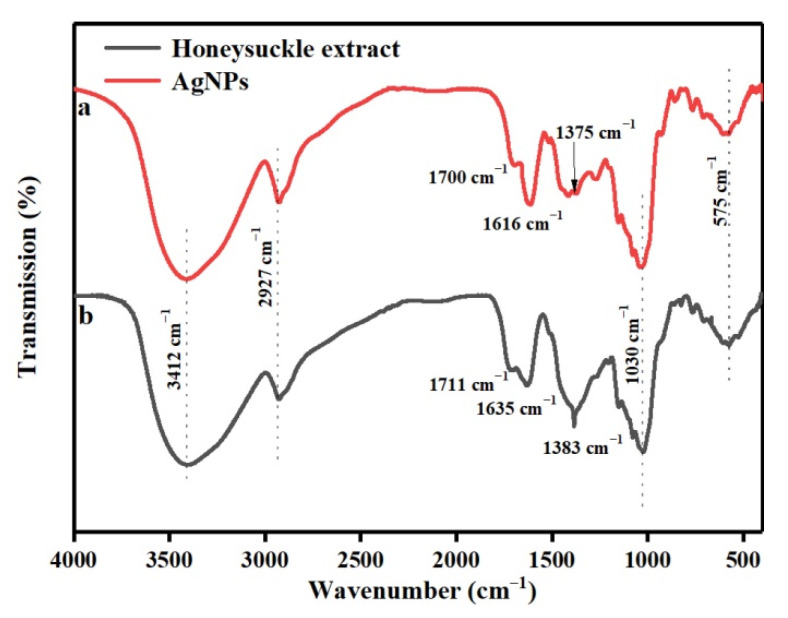
The FT-IR of Ag NPs (**a**) and Honeysuckle extract (**b**).

As shown in Figure 6, comparing the FT-IR spectra of the honeysuckle extract and the Ag NPs prepared using the honeysuckle extract, the change was not obvious. The strong absorption peak at 3412 cm^−1^ originates from the stretching vibration of the hydroxyl O–H bond in an alcohol or phenol, and the band at 2927 cm^−1^ corresponds to the C–H bond stretching of alkanes [30,31]. The bands at 1700 cm^−1^ and 1714 cm^−1^ correspond to the C=O bond stretching vibration of the carboxyl group of an aldehyde or ketone, the peaks at 1616 cm^−1^ and 1635 cm^−1^ correspond to the C=C stretching vibration, 1384 cm^−1^ and 1375 cm^−1^ are the absorption peaks of –CH3 bond stretching vibrations. The bending vibration of the –OH bond and the stretching vibration of the C–O bond are in the region of 1300–1000 cm^−1^ [32]. These results indicate that there were indeed some plant organic components on the surface of Ag NPs, which were likely to participate in the reduction of Ag^+^ and the stabilization of Ag NPs. Honeysuckle extract played an important role as a reducing agent and protective agent for preparing Ag NPs. Many chemicals in honeysuckle extract were attached to the surface.

### 3.6. Structure Characterization and Property Analysis of Ag NPs- Cotton Fabrics

#### 3.6.1. FT-IR Characterization of Ag NPs Finishing Cotton Fabric

**Figure 7:**
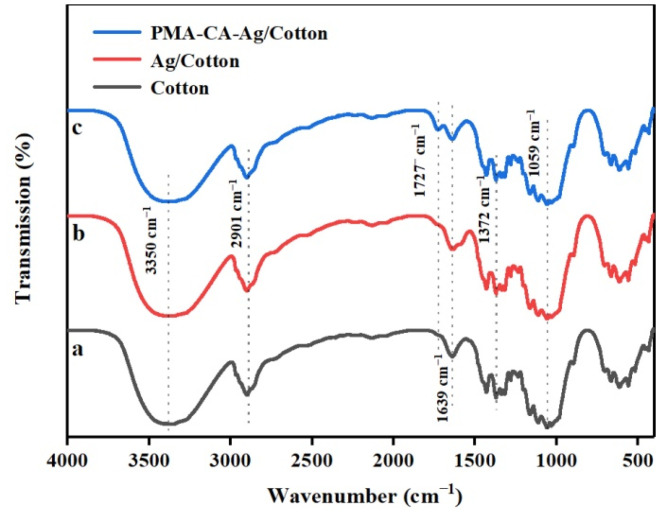
FT-IR image of Unfinished Cotton (**a**), Ag/Cotton (**b**) and PMA-CA-Ag/Cotton (**c**).

To verify that the cotton fabric was successfully cross-linked, the surface structure of the cotton fabric was characterized using infrared spectroscopy. The main component of cotton fiber is cellulose, and cellulose macromolecules have functional groups such as –CH2–, C–H, C–O, and O–H. It can be seen from the FT-IR spectrum of the unfinished cotton fabric in Figure 7a that the bands at 1372 cm^−1^ and 1059 cm^−1^ correspond to the bending vibration of –CH2– and the stretching vibration of C–O in cellulose, the absorption peak at 1639 cm^−1^ belongs to the bending vibration peak of water adsorption, 3350 cm^−1^ and 2901 cm^−1^ correspond to the O–H stretching vibration and C–H stretching vibration absorption peaks in cellulose, respectively [33]. It can be seen from the image of Ag/Cotton in Figure 7b, compared with the unfinished cotton, the infrared absorption band did not change much, indicating that the Ag NPs adsorbed on the fabric were not bonded to the fiber macromolecules, but were adsorbed on the fiber surface of cotton fabric by a van der Waals force, the infrared characteristic peak curve of cotton fiber fabric was not affected, and the chemical structure of cotton fabric was not changed. It can be seen from the infrared image of the PMA–CA–Ag/Cotton in Figure 7c, compared with the cotton fabric before cross-linking, the cross-linked cotton fabric showed a new absorption peak at 1727 cm^−1^, and because it is possible to confuse whether the peak is ester carbonyl or carboxyl carbonyl, the PMA–CA–Ag/Cotton was treated with 0.1 mol/L NaOH solution for 3 min at room temperature to convert unreacted carboxylic acid into carboxylate anion, so it corresponds to the absorption peak of the ester carbonyl group, which confirmed that carboxyl in the polycarboxylic acid and the hydroxyl group on the cellulose had an esterification reaction [34]. Therefore PMA + CA were cross-linked to cotton fabric.

#### 3.6.2. SEM Characterization of Ag NPs Finishing Cotton Fabric

**Figure 8:**
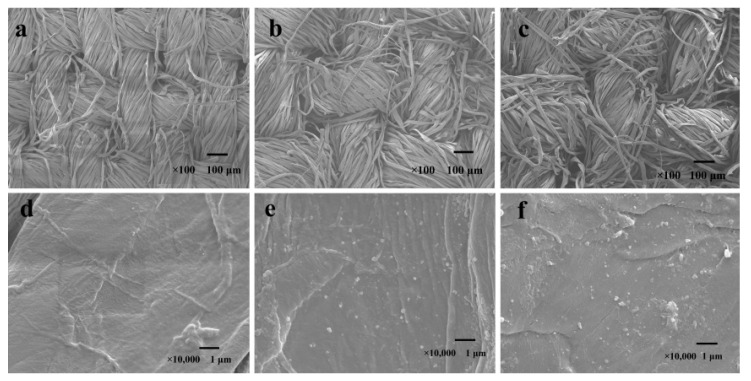
SEM images of Unfinished Cotton (**a**,**d**), Ag/Cotton (**b**,**e**) and PMA-CA-Ag/Cotton (**c**,**f**) were 100 and 10,000 times magnified.

From Figure 8a,d, there were many grooves on the surface of the unfinished cotton, and its appearance was rough, and it was not loaded with any granular material. As can be seen from Figure 8b,e, the surface of Ag/Cotton had obvious spherical particles, the average size of Ag NPs was smaller, and the distribution was more uniform.

It can be seen from Figure 8c,f that the cotton fabric was slightly damaged, and the roughness had increased. It may be that the cotton fiber was affected by the acid intolerance of cotton, but the particle size and distribution were still relatively uniform. It shows that the cross-linking of polycarboxylic acid had little effect on the particle size and distribution of Ag NPs. At the same time, it can be observed that the surface of the cotton fabric after finishing had slight Ag NPs agglomeration. The reason is that the Ag NPs were transferred from the finishing solution to the cotton fabric during the finishing process, and the dispersion medium had changed, and it is accompanied by rolling, drying, and other curing treatments. However, the particle size of most of the Ag NPs was relatively small and the distribution was still relatively uniform, indicating that the cross-linking of polycarboxylic acids had little effect on the particle size and distribution of the Ag NPs loaded on the cotton fabric

#### 3.6.3. XPS Characterization of Ag NPs Finishing Cotton Fabric

**Figure 9:**
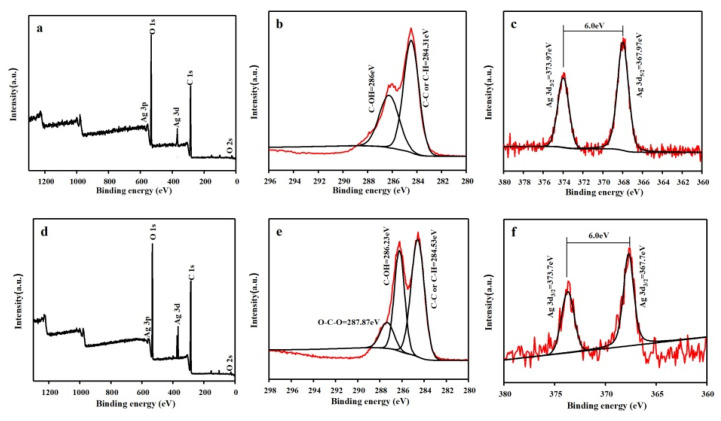
XPS spectra of Ag/Cotton (**a**–**c**) and PMA-CA-Ag/cotton (**d**–**f**).

To confirm further the success of the cross-linking of cotton fabric and determine the valence state of the Ag NPs loaded on the cotton fabrics, the C and Ag elements on the surface of the cotton fabric were analyzed using XPS. Figure 9 shows the XPS spectra of samples of Ag/Cotton and PMA–CA–Ag/Cotton. In Figure 9b, the C 1s spectral peak analysis shows that the binding energies of C–C or C–H and C–OH were 284.31 and 286 eV, respectively. In Figure 9e, the binding energies of C–C or C–H, C–OH, and O–C–O were 284.53, 286.23, and 287.87 eV, respectively. The appearance of the O–C–O peak indicates that the polycarboxylic acid was successfully grafted onto the cotton fabric. At the same time, the binding energy of Ag 3d in the finished cotton fabric of Ag NPs was analyzed using XPS to evaluate the chemical binding state of Ag in the cotton fabric. It can be seen from Figure 9a,d, that the binding energies were 368.16 eV. The strong Ag 3d signal peak is basically the same as the binding energy peak of pure silver element (368.2 eV) [35]. Similarly, in Figure 9c,f, through the analysis of the peak of the Ag 3d spectrum, the binding energies of Ag 3d_3/2_ and Ag 3d_5/2_ of the Ag/cotton were 373.97 and 367.97 eV, respectively. The binding energies of Ag 3d_3/2_ and Ag 3d_5/2_ of the PMA–CA–Ag/Cotton were 373.3 and 367.3 eV, respectively. The spin–orbit splitting of both samples was 6.0 eV. These data are completely consistent with the standard binding energy of Ag 3d. Therefore, the Ag loaded on the cotton fabrics was a simple substance [36,37]. Comparing the test results, the binding energy of C 1s and Ag 3d were basically unchanged, and the fluctuations between the values were within the allowable range of error.

#### 3.6.4. XRD Characterization of Ag NPs Finishing Cotton Fabric

**Figure 10:**
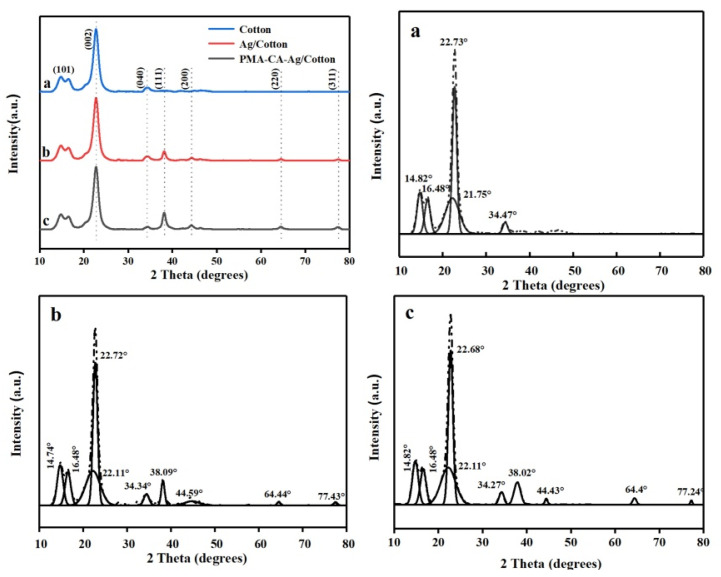
XRD peak-splitting fitting diagram of Unfinished Cotton (**a**), Ag/Cotton (**b**) and PMA-CA-Ag/Cotton (**c**).

To examine the change of crystallinity of cotton fabric before and after finishing, X-ray powder diffraction analysis was carried out on the unfinished fabric, Ag/Cotton, and PMA–CA–Ag/Cotton. From the XRD pattern of the unfinished fabric, it can be seen that the characteristic diffraction peaks of cellulose crystals appeared (2θ) at 14.82, 16.48, 22.73, and 34.47° [38]. Four new diffraction peaks appeared in both Ag/Cotton and PMA–CA–Ag/Cotton. The diffraction peaks of the crystal planes attributable to the Ag face-centered cubic crystal form are (111), (200), (220), and (311) [39,40], this is shown in Figure 10 (upper left). After fitting and decomposing the crystalline superimposed peaks and amorphous superimposed peaks of the diffraction curves of a, b and c in Figure 10 (upper left corner) with a Gaussian function, the crystallinity was calculated [41]. The calculated crystallinity of the unfinished cotton fabric was 69.71%, the crystallinity of Ag/Cotton was 72.41%, and the crystallinity of PMA–CA–Ag/Cotton was 74.31%, indicating that the crystallinity of the cotton fabric before and after finishing underwent little change. Therefore, the crystal structure of cotton fabric was not greatly affected by functional finishing.

#### 3.6.5. TG Characterization of the Ag NPs Cotton

**Figure 11:**
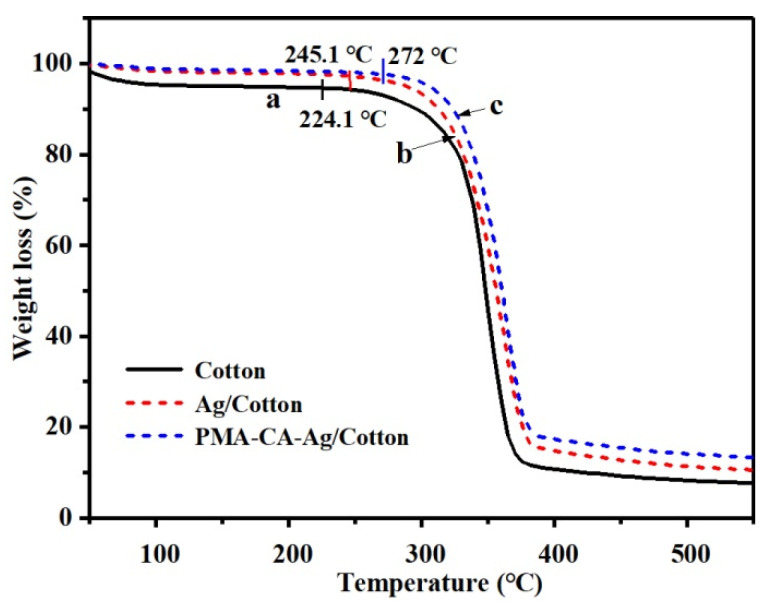
Thermogravimetric diagrams of Unfinished Cotton (**a**), Ag/Cotton (**b**) and PMA-CA-Ag/Cotton (**c**).

TGA was used to characterize the thermal properties of unfinished cotton, Ag/Cotton, and PMA–CA–Ag/Cotton (Figure 11).

As shown from Figure 11, the thermal decomposition starting temperature of unfinished cotton was 224.1 °C, and that of Ag/Cotton and PMA–CA–Ag/Cotton were 245.1 °C and 272 °C, respectively, which shows that the thermal stability of finished cotton fabric was better than unfinished cotton fabric. It can also be seen from Figure 11 that after the thermal degradation, the residual weight rate of the Ag/Cotton and PMA–CA–Ag/Cotton was 14.9% and 17.8%, respectively, which are slightly higher than the 10.6% residual weight rate of the unfinished cotton fabric. This part of the gap comes from the Ag NPs on the fiber that were not easily decomposed at high temperatures. In summary, the thermal stability of finished cotton fabrics was significantly higher than that of unfinished cotton.

#### 3.6.6. Antibacterial Property, Ag Content, and ∆E Test of Finished Cotton

##### Antibacterial and Washing Resistance Characterization

The prepared Ag NPs’ solution was diluted in a certain proportion and loaded on cotton fabric, and then the antibacterial properties, silver content, and color difference were measured.

**Figure 12:**
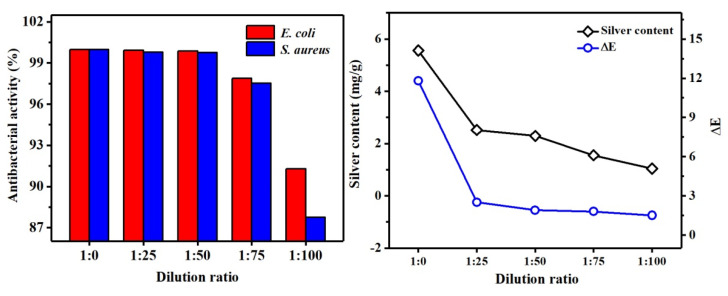
Under different dilution ratio of Ag/Cotton, the antibacterial rate (**left**), silver content and ∆E (**right**).

It can be seen from Figure 12 that as the dilution factor was increased, the concentration of Ag NPs in the solution was gradually decreased, so the content of silver loaded on the cotton fabric was gradually decreased. The data show that the antibacterial rates of 1:25 and 1:50 were basically the same. It shows that Ag NPs had good antibacterial properties, and that the fabric loaded with less silver content showed excellent antibacterial properties. Meanwhile, because Ag NPs are colored particles, when a certain amount of Ag NPs was adsorbed on the surface of the fabric, the fabric had a change in color, so there was a difference in color (∆E) compared with the unfinished cotton, and as shown from Figure 12, there was little difference between the ∆E of 1:25 and 1:50. To save costs and reduce the effect of Ag NPs on fabric color difference, the fabric with less silver content was selected, so the dilution factor was 1:50. Subsequent antibacterial test dilution times were subject to this.

##### Antibacterial and Washing Resistance Test

**Figure 13:**
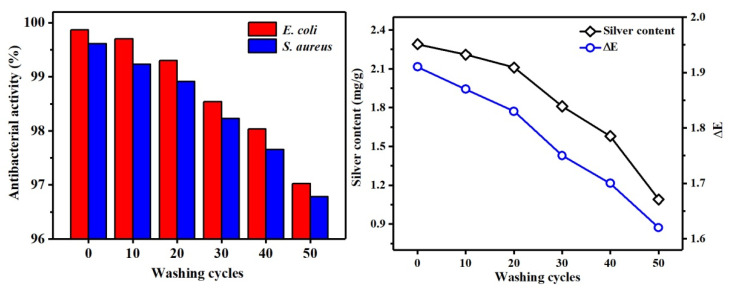
Distribution of antibacterial rate and antibacterial washing resistance of Ag/Cotton (**left**), distribution of silver content and ∆E (**right**).

**Figure 14:**
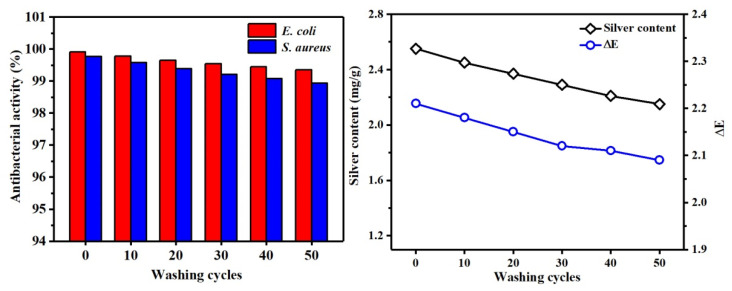
Distribution of antibacterial rate and antibacterial washing resistance of PMA-CA-Ag/Cotton (**left**); distribution of silver content and ∆E (**right**).

To test the antibacterial and washable performance of the fabric (Figure 13 and Figure 14), the Ag NPs’ solution prepared using honeysuckle extract was diluted 50 times to finish the cotton fabric, and then the antibacterial rate, silver content, and ∆E of the fabric after 50 washing cycles were tested. According to GB 15979-2002, before washing, the antibacterial rates of Ag/Cotton against *E. coli* and *S. aureus* were 99.87% and 99.61%, respectively, so the prepared cotton fabrics finished with honeysuckle extract Ag NPs reached the medical antibacterial level against *E. coli* and *S. aureus*. It can also be seen from Figure 13 (left) that the antibacterial rate decreased less before washing 20 times. This may be because Ag NPs were mainly physically adsorbed on the surface of the fabric and the interior of a large number of cavity structures. During the washing and friction, the Ag NPs might be shed by external force on the surface of the fabric, but the difference in the concentration of Ag NPs inside and outside the fabric allowed the internally adsorbed Ag NPs to be continuously added to the surface of the fabric, the decrease of the antibacterial rate of the fabric was small. However, after more than 20 washings, the antibacterial rate of the fabric decreased significantly. This may be because, after 20 washings, no internally adsorbed Ag NPs were transferred to the surface of the fabric, resulting in a continuous decrease in the number of Ag NPs physically adsorbed on the surface of the fabric, and the long-term antibacterial effect cannot be achieved. At the same time, it can be seen that the antibacterial performance of the finished cotton fabric against *E. coli* is better than that against *S. aureus*. The main reason is that the cell wall structures of *S. aureus* and *E. coli* are different. The cell wall of *E. coli* has a large number of negative charges, which can adsorb more Ag^+^ released by Ag NPs to penetrate the cell membrane and kill bacteria, whereas the cell wall of *S. aureus* has a large number of positive charges, which can cause certain obstacles for Ag^+^ to enter the cell membrane, so it has a lower antibacterial ability [42].

It can be seen from Figure 13 (right) that the silver content and ∆E of Ag/Cotton were 2.29 mg/g and 1.91, respectively. As the number of washing times increased, the silver content on the fabric gradually decreased, and the amount of Ag NPs adsorbed on the surface gradually decreased, resulting in a lighter surface color.

It can be first observed from Figure 14 (left) that the antibacterial rates against *E. coli* and *S. aureus* were 99.91% and 99.77%, respectively. After 50 washings, the antibacterial rate of PMA–CA–Ag/Cotton on the two strains did not decrease significantly, and it still had good antibacterial and washing resistance. The principle was through esterification, PMA + CA was fixed to cotton fabric, Ag NPs were positively charged in acid solution, and the carboxylic anion of polycarboxylic acid had a strong chelating ability, so the Ag NPs could easily coordinate with the O atoms in the unesterified carboxyl group in PMA + CA, and were loaded onto the fiber surface through chelation. The PMA + CA played a bridging role between Ag NPs and cotton fabric, that is, chemical bond formation [43,44]. In addition, some of the Ag NPs might be directly loaded on the cotton fabric through van der Waals forces. Therefore, the combination of two forces between Ag NPs and the fabric can help to improve the load firmness of the cotton fabric to Ag NPs, and can achieve long-term antibacterial effects. From the comparison of Figure 13 and Figure 14, it can be seen that PMA–CA–Ag/Cotton had a slightly higher antibacterial rate against *E. coli* and *S. aureus* than Ag/Cotton. The reason may be that the Ag NPs on PMA-CA-Ag/Cotton had not only van der Waals force but also a chemical bond between the cotton fabric, which could adsorb more Ag NPs. 

As can be seen from Figure 14 (right), when the PMA–CA–Ag/Cotton was not washed, the value of silver content and ∆E were 2.55 mg/g and 2.21, respectively. This is because the cotton fabric was cross-linked by polycarboxylic acid, thus becoming slightly yellow, which increased the ∆E on the surface of the fabric compared with Ag/Cotton. However, as washing cycles increased, the ∆E of cotton fabric did not change much. It may be that the Ag NPs bound by chemical bonds and physical bonding were relatively stable.

#### 3.6.7. UV and Wrinkle Resistance Characterization

It can be seen from Table 1 that compared with the unfinished sample, the average ultraviolet transmittance of the UVA (315–400 nm) and UVB (280–315 nm) segments of Ag/Cotton and PMA–CA–Ag/Cotton were significantly reduced. The UPF value increased significantly, indicating that the load of Ag NPs greatly improved the UV resistance of cotton fabric. According to the requirements of GB/T18830-2002, when the UPF > 30 and UVA < 5%, it is an anti-ultraviolet product, and the mark is 30+. It can be seen that Ag/Cotton and PMA–CA–Ag/Cotton can be called UV-resistant textiles. This may be because when the fabrics finished with Ag NPs were irradiated with ultraviolet rays, the reflection and scattering of ultraviolet rays on the surface of the fabrics were increased through the interface formed by the metal ion and the fabric, so that the ultraviolet rays were shielded. At the same time, because of the small size of Ag NPs and the quantum size effect, as UV was absorbed, the UV resistance of cotton fabrics was improved. The cross-linked structure of PMA–CA–Ag/Cotton made the fabric dense, ultraviolet rays could be effectively blocked, and the pass rate of ultraviolet rays was reduced. It can be seen that the UV protection function of cotton fabric was improved by Ag NPs finishing technology to a certain extent.

As shown From Table 1, compared with unfinished cotton, the WRA of Ag/Cotton was increased by about 4.34%, the WRA of PMA–CA–Ag/Cotton was increased by about 71.01%, which was about 63.89% higher than that of Ag/cotton. Cellulose macromolecules were connected by glucose rings, and the protruding hydroxyl groups on the chain provide reactive cross-linking sites [45]. The cross-linking sites of the cellulose were cross-linked with the hydroxyl group in the accessible area of the cellulose to form a cross-linked network structure on the surface of the cotton fabric, which prevented the slippage of the fiber macromolecular chain, and gave the cellulose better antideformation ability, and improved the elastic recovery ability after deformation [46]. 

## 4. Conclusions

In this paper, honeysuckle extract was used as a reducing agent and protective agent to prepare high-purity Ag NPs, with a particle size of about 10.59 nm, with good stability, and strong electrostatic repulsion. After the Ag NPs were loaded on the fabric by the dipping method, Ag/Cotton was obtained, and then PMA + CA was cross-linked to the Ag NPs’ cotton fabric by the dipping method to obtain PMA–CA–Ag/Cotton. Through structural characterization and performance analysis, we found that PMA + CA and Ag NPs were covalently bonded together without damaging the structure of the fabric. The surface of the fabric was still loaded with Ag NPs without oxidation, indicating that the stability of the synthesized Ag NPs was good, the thermal stability of PMA–CA–Ag/Cotton was improved, and at the same time, it gave the fabric long-term antibacterial properties, and had little effect on the color of the fabric. The wrinkle resistance and UV resistance performances of the cross-linked Ag NPs cotton fabric were greatly improved.

## Figures and Tables

**Table 1:** UV and wrinkle resistance of the Unfinished Cotton, Ag/Cotton and PMA-CA-Ag/Cotton.

Sample	Average UV Transmittance (%)		UPF	WRA(W + F(°))
	UVA	UVB		
Unfinished Cotton	29.98	15.65	5.36	138
Ag/Cotton	1.8	2.76	36.31	144
PMA-CA-Ag/Cotton	2.05	2.52	39.45	236

## Data Availability

The data presented in this study are available on request from the corresponding author.
